# Editorial: Evolution of crop genomes and epigenomes

**DOI:** 10.3389/fpls.2022.1027698

**Published:** 2022-09-22

**Authors:** Hai Du, Wei Hu, Viktor Demko, Zhe Liang

**Affiliations:** ^1^ College of Agronomy and Biotechnology, Chongqing Engineering Research Center for Rapeseed, Southwest University, Chongqing, China; ^2^ Integrative Science Center of Germplasm Creation in Western China (CHONGQING) Science City and Southwest University, College of Agronomy and Biotechnology, Southwest University, Chongqing, China; ^3^ Engineering Research Center of South Upland Agriculture, Ministry of Education, Chongqing, China; ^4^ Institute of Tropical Bioscience and Biotechnology, Chinese Academy of Tropical Agricultural Sciences, Haikou, China; ^5^ Faculty of Natural Sciences, Comenius University in Bratislava, Bratislava, Slovakia; ^6^ Plant Science and Biodiversity Center, Slovak Academy of Sciences, Bratislava, Slovakia; ^7^ Biotechnology Research Institute, Chinese Academy of Agricultural Sciences, Beijing, China

**Keywords:** multi-omics, crops, genome, epigenome, evolution, epigenomics

In the last two decades, the advances in large-scale sequencing technology (e.g., the second- and third-generation sequencing technologies) have generated increasingly available omics datasets (e.g. genomics, chloroplast, epigenomics, transcriptomics) of plant organisms, offering valuable resource to address the various biological questions more effectively. Available genome resources allow us to uncover yet hidden mysteries of plant genomes and their evolutionary stories at genome-wide level and to screen for agronomically significant genes in crops. In this Research Topic, we aim to present novel and important findings on all aspect of genome and epigenetics in plants, especially crops. This topic includes 12 original research papers focusing on the research areas highlighted above, viewed more than 15,263 times by the time of this Editorial. We organize this collection of studies into two major groups based on common themes as described below.

## Genome-wide evolution analysis and gene resource screen

Hu et al. present a systematic identification and evolutionary analysis of the multiple C2 domain and transmembrane region proteins (MCTPs) in upland cotton, and also conducted a phylogenetic analysis with the homologs in another 17 plant species including algae, fern, moss, monocotyledons and dicotyledons. Based on global expression analyses using transcriptome dataset and qRT-PCR assay, they identify three candidate genes (*GhMCTP7*, *GhMCTP12* and *GhMCTP17*) and demonstrate the interaction of GhMCTP7/12/17 with GhKNAT1/2 proteins to regulate cotton shoot meristem development in an integrated multiple signal pathway manner.

Sun et al. conducted a genome-wide study on the plant-specific SQUAMOSA promoter-binding protein-like (SPL) transcription factor family in four *Ipomoea* species, including gene and protein structure characteristics, gene duplication, selective pressure, expansion pattern, spatiotemporal and exogenous phytohormone induction expression profiles, etc. This study revealed that segmental duplication is the main driving force for gene expansion in *Ipomoea* species. The data suggest that most of the *Ipomoea SPL* genes are miR156 targets with seven miR156-SPL interaction relationships were verified by degradome sequencing. They finally identify four *SPL* genes with putative function in sweet potato storage root development.

Li et al. identified and analyzed the K^+^ efflux antiporter (KEA) genes in four cotton species and seven other plants (*Arabidopsis thaliana*, *Oryza sativa*, *Zea mays*, *Populus trichocarpa*, *Sorghum bicolor*, *Triticum aestivum* and *Glycine max*) at genome-wide level. The candidate genes were classified into three subfamilies with similar motif compositions and gene structure characteristics in each family. They found that segmental replication and purifying selection play key role in the evolution of the *KEA* gene family in upland cotton. The global expression profiles of *KEA* genes in various tissues and under multiple stress conditions (e.g. salt, drought, and low potassium treatments) were comprehensively analyzed in upland cotton. Virus-induced gene silencing (VIGS) experiment demonstrated that two candidate genes (*GhKEA4* and *GhKEA12*) are involved in salt and potassium stresses response in upland cotton.

Song et al. performed an in-depth investigation on the slow type anion channels (SLAHs) gene family in Cassava, including phylogenetic relationship with other related organisms, chromosomal localization and genome-wide expression analysis. *MeSLAH4* gene was identified as a potential nitrogen-responsive gene, and overexpression analysis in rice demonstrated its capability to enhance the nitrogen assimilation, root growth, and grain yield, indicating its vital role in enhancing nitrogen utilization efficiency and yield.

Peng et al. investigated the three-amino-acid-loop-extension (TALE) transcription factor encoding genes in sweet orange genome, accompanied by systematic analysis regarding their phylogeny, evolution, gene and protein structure, *cis*-acting regulatory element, and protein–protein interaction. They revealed that segmental duplication and purifying selection are the major driving force in the evolution of this gene family in sweet orange. The biological functions of this gene family in sweet orange in response to biotic and abiotic stresses were elucidated by global biotic/abiotic- stress-induced expression pattern analyses (e.g. high temperature, salt, wounding and pathogen stresses). Then, the authors confirmed the transcriptional activity and protein interaction networks of several candidate TALE proteins using yeast two-hybridization assay system

Xie et al. performed a genome-wide analyses of the serine/arginine-rich (SR) gene family in *Brassica napus*, and demonstrated that the genes in each subfamily have conserved structures and motifs and distinct expression patterns. They found that this gene family had a widespread alternative splicing pattern including the paralogous gene pairs. They identified 12 *SR* genes that were potentially associated with specific agronomic traits in *B. napus* by association mapping analysis.

Huang et al. generated a moso bamboo transcriptome dataset under dehydration and cold treatments using RNA-seq technology. This study identified a series of differentially expressed genes and dehydration- and cold-responsive genes. The authors selected a dehydration-responsive gene, *PeLEA14*, as the candidate gene, and proved the roles of *PeLEA14* in response to abiotic stress tolerance including salt stress by overexpression analysis in tobacco.

Li et al. performed genome-wide association study for drought-resistance traits in hulless barley. They evaluated various quantitative traits and field phenotypes of 269 hulless barley lines under either normal or drought conditions. They obtained a total of 8,936,130 highly consistent population SNP markers. Eight candidate genes were suggested to be involved in drought resistance in this genus.

## Chloroplast genome sequencing and evolution analysis

Fan et al. reported the chloroplast (cp) genomes of three *Fagopyrum* species, and presented an integrated analysis with five published *Fagopyrum* cp genomes. The evidence of sequence differentiation, repeated sequences, gene number, gene order, codon usage and phylogenetic relationship were presented. This study detected six variable regions and 66 SSR types in the eight species with potential applications in plant genetic relationship and taxonomic status identification. They supported the unique taxonomic status in *Fagopyrum* in the Polygonaceae.

Song et al. assembled and compared the complete cp genomes of nine new *Musa* plants with focus on the genomic features and phylogenetic implications. They found that the studied *Musa* chloroplast genomes commonly encoded 135 functional genes that were composed of photosynthesis-related genes, chloroplast self-replication genes, and other genes. This study identified six non-coding sites and three genes that can be used for DNA barcoding and phylogenetic analysis. They divided the nine *Musa* species into two groups based on phylogenetic analyses.

Li et al. provided 27 complete cp genomes of 11 wild *Fragaria* species accompanied by in-depth variation and evolutionary analyses. They found that the genome structure is highly conserved among these cp genomes, and non-coding regions were relative more variable than coding regions. In addition, the authors revealed that the contraction and expansion of the inverted repeat (IR) regions contributed to different cp genome size in these *Fragaria* species. Five variable loci that could be developed as DNA barcoding for *Fragaria* species were identified. The authors divided the studied species into two groups: Group A distributed in western China and Group B originating from Europe and Americas. The results also revealed allopolyploid origins of the octoploid and tetraploid *Fragaria* species.

Tian et al., present plastome sequencing of 33 peanuts, and then explore their taxonomic status and evolutionary relationship. The authors divided the studied species into two lineages: Lineage I containing all the cultivated species and Lineage II possessing diverse genome types. Next, the authors suggested that all allotetraploid cultivated peanut species were derived from a maternal hybridization event with one of the diploid *Arachis duranens* accessions having a AA sub-genome ancestor, and *Arachis monticola* that represent transitional tetraploid wild species of all the cultivated peanuts.

## Author contributions

HD wrote the first draft of the editorial. ZL, WH, and VD all commented on the draft. All authors contributed to the editorial and approved it for publication.

## Funding

This work was supported by the National Natural Science Foundation of China (32072094), the Major Science and Technology Program of Hainan Province (ZDKJ202001), the Central Public-interest Scientific Institution Basal Research Fund for Chinese Academy of Tropical Agricultural Sciences (1630052022017 and 1630052020006), and Slovak Research and Development Agency grant APVV-17-0570.

## Acknowledgments

The editors would like to thank all the authors and reviewers who have participated in this Research Topic.

## Conflict of interest

The authors declare that the research was conducted in the absence of any commercial or financial relationships that could be construed as a potential conflict of interest.

## Publisher’s note

All claims expressed in this article are solely those of the authors and do not necessarily represent those of their affiliated organizations, or those of the publisher, the editors and the reviewers. Any product that may be evaluated in this article, or claim that may be made by its manufacturer, is not guaranteed or endorsed by the publisher.

